# Elevated CO_2_ and Warming Altered Grassland Microbial Communities in Soil Top-Layers

**DOI:** 10.3389/fmicb.2018.01790

**Published:** 2018-08-14

**Authors:** Hao Yu, Ye Deng, Zhili He, Joy D. Van Nostrand, Shang Wang, Decai Jin, Aijie Wang, Liyou Wu, Daohan Wang, Xin Tai, Jizhong Zhou

**Affiliations:** ^1^Key Laboratory of Environmental Biotechnology, Research Center for Eco-Environmental Sciences, Chinese Academy of Sciences (CAS), Beijing, China; ^2^College of Environmental Science and Engineering, Liaoning Technical University, Fuxin, China; ^3^College of Resources and Environment, University of Chinese Academy of Sciences, Beijing, China; ^4^Environmental Microbiome Research Center, School of Environmental Science and Engineering, Sun Yat-sen University, Guangzhou, China; ^5^Institute for Environmental Genomics, The University of Oklahoma, Norman, OK, United States; ^6^State Key Laboratory of Urban Water Resource and Environment, Harbin Institute of Technology, Harbin, China; ^7^State Key Joint Laboratory of Environment Simulation and Pollution Control, School of Environment, Tsinghua University, Beijing, China

**Keywords:** elevated carbon dioxide, warming, soil microbial community, Prairie Heating and CO_2_ Enrichment (PHACE) experiment, functional genes, grassland ecosystem

## Abstract

As two central issues of global climate change, the continuous increase of both atmospheric CO_2_ concentrations and global temperature has profound effects on various terrestrial ecosystems. Microbial communities play pivotal roles in these ecosystems by responding to environmental changes through regulation of soil biogeochemical processes. However, little is known about the effect of elevated CO_2_ (eCO_2_) and global warming on soil microbial communities, especially in semiarid zones. We used a functional gene array (GeoChip 3.0) to measure the functional gene composition, structure, and metabolic potential of soil microbial communities under warming, eCO_2_, and eCO_2_ + warming conditions in a semiarid grassland. The results showed that the composition and structure of microbial communities was dramatically altered by multiple climate factors, including elevated CO_2_ and increased temperature. Key functional genes, those involved in carbon (C) degradation and fixation, methane metabolism, nitrogen (N) fixation, denitrification and N mineralization, were all stimulated under eCO_2_, while those genes involved in denitrification and ammonification were inhibited under warming alone. The interaction effects of eCO_2_ and warming on soil functional processes were similar to eCO_2_ alone, whereas some genes involved in recalcitrant C degradation showed no significant changes. In addition, canonical correspondence analysis and Mantel test results suggested that NO_3_-N and moisture significantly correlated with variations in microbial functional genes. Overall, this study revealed the possible feedback of soil microbial communities to multiple climate change factors by the suppression of N cycling under warming, and enhancement of C and N cycling processes under either eCO_2_ alone or in interaction with warming. These findings may enhance our understanding of semiarid grassland ecosystem responses to integrated factors of global climate change.

## Introduction

With the rapid and continuous increase in fossil fuel emissions since the beginning of the Industrial Revolution, the concentration of atmospheric CO_2_ has risen sharply from 280 to 406.53 ppm in 2017 (Ruddiman, 2013; Pieter Tans, 2017). The high levels of CO_2_ and other greenhouse gasses have led to an increased global temperature and reduced precipitation (IPCC, 2014). Soil microbial communities (i.e., bacteria, archaea and fungi) are regarded as sensitive indicators of soil quality and are responsible for belowground carbon (C) and nutrient cycling in various ecosystems. The community structure and functional processes can be influenced by temperature and elevated CO_2_ (eCO_2_) both directly and indirectly via biotic and abiotic factors, such as soil C inputs, moisture and temperature (Castro et al., 2010). Therefore, it is crucial to understand the combined effect of eCO_2_ and warming on the functional diversity, composition, structure and dynamics of soil microbial communities and their correlations with ecosystem processes.

Grass-dominated terrestrial ecosystems contain more than 10% of the global carbon (C) stock and account for over 30% of the global aboveground net primary production (NPP) (Jones and Donnelly, 2004; Grosso et al., 2008), and also provides the majority of forage for feeding livestock. The priming effects of CO_2_ in grasslands are well known and include increased above- and belowground plant biomass, photosynthetic C allocation to roots, belowground C inputs and rhizodeposition (Pendall et al., 2004; Carol Adair et al., 2009; Adair et al., 2011; Carrillo et al., 2011; Lee et al., 2011). The combination of eCO_2_ with warming and warming alone showed uncertain effects on above- and belowground production, C allocation, and the soil nitrogen (N) status, which may be highly correlated with soil water availability (Dijkstra et al., 2010, 2013a; Carrillo et al., 2011; Morgan et al., 2011). However, how eCO_2_ and warming, particularly when combined, impact the functional diversity, composition, structure and functional processes of soil microbial communities are still unclear in water-constrained grasslands. For example, warming may increase biomass and microbial activity in a prairie ecosystem (Belay-Tedla et al., 2009), but the pattern may be altered under water limited conditions or reduced soil C inputs (Castro et al., 2010). Rising CO_2_ may increase soil water availability, improving plant water-use efficiency (Wan et al., 2007; Leakey, 2009), but this effect may be offset by warming-induce desiccation in water-constrained ecosystems (Morgan et al., 2011). The effect of CO_2_ and temperature on soil C may be mediated by the impact of these variables on soil water availability via regulation of decomposition and plant inputs in semiarid grassland ecosystems (Carrillo et al., 2011), which in turn alters the composition, structure and functional processes of microbial communities. However, the interactive effects of multiple global change factors (e.g., eCO_2_, warming, elevated O_3_ and precipitation) on soil microbial communities had been less well studied (Castro et al., 2010). Therefore, a comprehensive evaluation of the effect of warming and eCO_2_ on soil microbial communities, especially in water limited ecosystems, is necessary.

To model the effects of eCO_2_. and warming, a Prairie Heating and CO_2_ Enrichment (PHACE) experiment was conducted on semiarid temperate mixed grass prairies in Wyoming, United States (Parton et al., 2007). The gross primary production, root biomass, ecosystem respiration, soil organic carbon, net soil nitrogen (N) release and mineralization associated with soil moisture were altered under multiple factor conditions (Dijkstra et al., 2010; Carrillo et al., 2011, 2012; Ryan et al., 2015, 2017; Mueller et al., 2016). For example, a previous study showed that eCO_2_ significantly decreased soil inorganic N due to the increase of microbial N immobilization, and warming significantly increased soil inorganic N and plant N pool sizes, while the combined effects of eCO_2_ and warming on N pool sizes were not significant (Dijkstra et al., 2010). These changes may directly or indirectly affect the structure and functional processes (e.g., C and N cycling) of the soil microbial community.

A high-throughput functional gene array (GeoChip 3.0) (He et al., 2010a) was employed to analyze the soil microbial communities in the above mentioned semiarid grassland experimental site. GeoChip 3.0 contains approximately 28,000 oligonucleotide probes involved in many biogeochemical functional processes [such as C, N, sulfur (S) and phosphorus (P) cycling], and has been used to examine the microbial communities from various environments (Yu et al., 2014a; Cai et al., 2015; Xiong et al., 2015; Xue et al., 2016a; Yu et al., 2018). In this study, we attempted to address whether (i) the functional composition and structure of soil microbial communities would be dramatically altered as soil C inputs and soil properties change in response to multiple climate factors; (ii) soil microbial functional processes (e.g., C and N cycling) would have different responses to warming, eCO_2_ and the interaction between these two factors. This study has important implications for soil microbial communities in response to global climate changes in grassland ecosystems.

## Materials and Methods

### Site Description and Sampling

The PHACE experiment was conducted at the United States Department of Agriculture’s Agricultural Research Service (USDA-ARS) High Plains Grasslands Research Station in Cheyenne, WY, United States (latitude 41°11′N, longitude 104° 54′W). The ecosystem is dominated by two C3 grasses, *Hesperostipa comata* Trin and Rupr. and *Pascopyrum smithii* (Rydb.) and a C4 grass, *Bouteloua gracilis* (H.B.K.) Lag. The average annual precipitation is 388 mm (Zelikova et al., 2014), and the mean air temperature is -2.5°C in winter and 17.5°C in summer. The soil at the experimental site is a fine-loamy, mixed, mesic Aridic Argiustoll (Morgan et al., 2011).

Twenty 3.4 m diameter circular plots were constructed with a 60 cm deep impermeable barrier. The PHACE experiment was conducted in a full factorial design to evaluate the combined effect of CO_2_ and temperature with five replicates per treatment. Plots were randomly assigned to four treatments including two concentrations of CO_2_ treatment (ambient vs. 600 μmol mol^-1^) since 2006, and two levels of warming treatment [ambient vs. warming of the canopy above ambient (+1.5°C, day; +3.0°C, night)] since 2007: (i) ambient, ambient CO_2_ and ambient temperature; (ii) warming, ambient CO_2_ and elevated temperature; (iii) eCO_2_, elevated CO_2_ and ambient temperature; (iv) eCO_2_ + warming, elevated CO_2_ and elevated temperature. Warming and Free Air CO_2_ Enrichment (FACE) technology was used as previously reported (Dijkstra et al., 2010; Morgan et al., 2011).

Five replicate samples were collected form each treatment plot (ambient, warming, eCO_2_, eCO_2_ + warming) at a soil depth of 0–5 cm in 2008. After the removal of plant residual roots and rocks, all PHACE soil samples were immediately stored at -80°C or 4°C for DNA extraction and soil property analysis, respectively.

### Soil Property Analysis

Soil total carbon (TC) and nitrogen (TN) were measured by dry combustion using a Leco TruSpec carbon and nitrogen analyzer. The NO_3_-N and NH_4_-N were extracted from soil samples by the use of 1 M KCl solution and quantified by a Lachat Quickchem 8500 series 2 instrument (Lachat, Loveland, CO, United States). Soil pH was measured using a glass electrode in a 1:2.5 (soil:water) solution (w/v).

### DNA Extraction and GeoChip Analysis

Soil DNA was extracted from 5 g soil samples using a freeze-grinding method (Zhou et al., 1996) and was purified using a Promega Wizard DNA clean-up system (Madison, WI, United States). DNA quality was measured using an ND-1000 spectrophotometer (NanoDrop Technologies Inc., Wilmington, NC, United States) to determine 260/280 nm and 260/230 nm ratios, and DNA concentration was quantified with Quant-It PicoGreen (Invitrogen, Carlsbad, CA, United States). Approximately 3 μg purified DNA per sample was labeled with the fluorescent dye Cy-5 (GE Healthcare) using a random priming method (He et al., 2014; Yu et al., 2014b, 2018).

Hybridizations were performed with the GeoChip 3.0 on a MAUI hybridization system (Biomicro Systems, Salt Lake City, UT, United States) at 42°C and 40% formamide for 12 h. After washing and drying, GeoChip slides were scanned by a ProScan array microarray scanner (PerkinElmer, Boston, MA) (Xue et al., 2016b) at a laser power of 95% and a photomultiplier tube (PMT) gain of 75%, and the images were quantified using ImaGene 6.0 (Biodiscovery, El Segundo, CA, United States) to determine the intensity of each spot.

Poor-quality spots with a signal-to-noise ratio (SNR) (SNR = [signal mean - background mean]/background standard deviation) of >2.0 were removed as previously described (He and Zhou, 2008). After removal of poor-quality spots, the signal intensities of the probes were normalized within and across all samples on our microarray processing pipeline^1^ (He et al., 2010a; Liang et al., 2010). Those gene probes that were detected in at least two of the 5 replicate samples were considered positive, and data can be found on our website^2^.

### Statistical Analysis

Significant changes in soil properties between ambient and warming or eCO_2_ and eCO_2_ + warming were determined by unpaired *t*-tests and analysis of variance (ANOVA). The overall changes in microbial functional and phylogenetic structure were determined by detrended correspondence analysis (DCA) and permutational multivariate analysis of variance (Adonis). The significant differences in individual genes between ambient and the three treatments were calculated by unpaired *t*-tests. The correlation between the microbial functional structure and soil properties was analyzed by canonical correspondence analysis (CCA) and Mantel test. All statistical analyses were performed by R project v.3.2.1 ^3^ using the Vegan and Agricolae package.

## Results

### Effects of Warming, eCO_2_, eCO_2_ + Warming on Soil Properties

Soil parameters showed different trends under warming, eCO_2_, and eCO_2_ + warming treatments (**Table 1**). First, NO_3_-N was significantly lower (*P* < 0.05, *t*-test) under eCO_2_ and eCO_2_ + warming conditions compared with control, while there were no significant differences between ambient and warming. Second, NH_4_-N was significantly lower (*P* < 0.05, *t*-test) under eCO_2_ than ambient but the difference was not significant between ambient and warming or eCO_2_ + warming. Third, soil moisture was significantly lower (*P* < 0.05, *t*-test) under warming than ambient, but was higher at significant (*P* < 0.05, *t*-test) and marginal (*P* < 0.1, *t*-test) levels under eCO_2_ and eCO_2_ + warming than ambient, respectively. Fourth, no significant differences were observed in TN, TC, C/N ratio and pH between ambient and warming, or eCO_2_, and eCO_2_ + warming. These results indicated that eCO_2_ significantly affected soil NO_3_-N, NH_4_-N and moisture, while warming and eCO_2_ + warming significantly affected only soil moisture and NO_3_-N, respectively.

**Table 1:** Effects of warming, eCO_2_ and eCO_2_ + Warming on soil properties.

	NO_3_-N (mg/kg)	NH_4_-N (mg/kg)	TN (%)	TC (%)	C/N	Moisture (%)	pH
Warming effect*^a^*	0.860	0.536	0.001	-0.033	-0.183	**-1.255^∗^**	-0.145
eCO_2_ effect	**-2.136^∗∗b^**	**-0.759^∗^**	-0.019	-0.198	0.003	**1.980^∗^**	-0.134
eCO_2_ + Warming effect	**-1.476^∗^**	-0.034	-0.024	-0.194	0.357	1.072^∙^	-0.033


*TN, total nitrogen; TC, total carbon; C/N, TC/TN ratio; ANOVA, analysis of variance; *^*a*^*Soil property values were analyzed and represented with differences of mean (treatment – ambient). *^*b*^*The significance of treatment effects were analyzed by *t*-tests. Significant differences (*P* < 0.05) indicated by bold type. Asterisks denote the *P*-value for the difference: ^∗∗^*P* ≤ 0.01, ^∗^*P* ≤ 0.05, ^∙^*P* < 0.1.*

### Effects of Warming, eCO_2_, eCO_2_ + Warming on Functional and Phylogenetic Structure of Soil Microbial Communities

A total of 3,624 microbial function genes were detected under four treatments across 20 samples. A significantly (*P* < 0.05) greater number of genes were detected under eCO_2_ (2,217 ± 269) than ambient (1,269 ± 78) (**Supplementary Table S1**), but the difference was not significant between ambient and either warming or eCO_2_ + warming. Analysis of alpha-diversity indexes showed similar patterns. eCO_2_ significantly (*P* < 0.05) increased the Shannon index (H′) and the Simpson’s reciprocal index (1/D) compared to ambient, but no significant differences were found between ambient and other treatments. The overall taxonomic composition of soil microbial community under different treatments was further analyzed at phylum level based on GeoChip data (**Supplementary Figure S1**). The detected functional genes were taxonomically derived from 2 archaeal phyla, 17 bacterial phyla, and 3 eukaryotic phyla. Proteobacteria (69.45% – 66.13%), Actinobacteria (17.78% – 13.68%), Firmicutes (4.41% – 3%), Ascomycota (4.35% – 2.69%) and Chloroflexi (2.02% – 1.11%) were detected as the five dominant phyla. eCO_2_ and eCO_2_ + warming significantly impacted the abundance of key genes derived from these five dominant phyla (**Supplementary Figure S2**).

**FIGURE 1:**
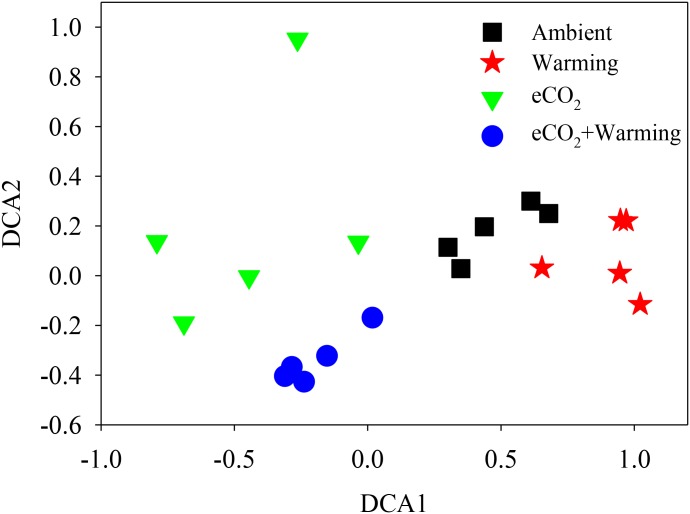
Detrended correspondence analysis (DCA) of all detected functional genes across three treatments and the ambient samples.

The Adonis test of all detected genes showed that eCO_2_, warming, and their combined effect significantly (*P* < 0.05) impacted soil microbial communities (**Table 2**). About 41.1% of the total variation can be explained by this model with eCO_2_ (26.4%) as the main factor, followed by warming (7.6%) and eCO_2_ + warming (7.1%). Moreover, the soil microbial phylogenetic structure based on the analysis of *gyrB*, a phylogenetic marker gene, was significantly (*P* < 0.05) influenced by all treatments (eCO_2_, 24.5%; warming, 6.9%; eCO_2_ + warming, 7.9%) (**Table 2**). Detrended correspondence analysis of all detected functional genes and of *gyrB* genes indicated that samples from the four treatment plots were distinct from each other (**Figure 1** and **Supplementary Figure S3**). These results indicated that the diversity, composition, and phylogenetic and functional gene structures of the soil microbial communities was changed under eCO_2_, warming and eCO_2_ + warming treatments in semiarid grassland.

**Table 2:** Adonis analysis of the effect of eCO_2_, Warming and eCO_2_ + Warming on the functional and phylogenetic structure of microbial communities based on all detected genes and *gyrB* genes, respectively.

	eCO_2_		Warming		eCO_2_ + Warming	
	R2	*P*	R2	*P*	R2	*P*
Functional structure	0.264	**0.001^∗∗∗^**	0.076	**0.030^∗^**	0.071	**0.047^∗^**
Phylogenetic structure (*gyrB*)	0.245	**0.001^∗∗∗^**	0.069	0.056^∙^	0.079	**0.042^∗^**


*Asterisks denote the P-value for the difference: ^∗∗∗^*P* ≤ 0.001, ^∗∗^*P* ≤ 0.01, ^∗^*P* ≤ 0.05, ^∙^*P* ≤ 0.1.*

### Effects of Warming, eCO_2_, eCO_2_ + Warming on Key Functional Genes Involved in Major Biochemical Process

A total of 138 ± 9, 94 ± 12, 245 ± 26, and 200 ± 9 genes involved in C cycling (including C fixation, degradation and methane metabolism) showed positive signals under ambient, eCO_2_, warming and eCO_2_ + warming treatments, respectively. Compared with ambient, detected gene numbers were significantly (*P* < 0.05) higher in the samples from eCO_2_ treatments.

Two key carbon fixation genes were detected, including Pcc (propionyl-CoA carboxylase) and Rubisco (ribulose-1,5-bisphosphate carboxylase/oxygenase) (**Supplementary Figure S4**). Elevated CO_2_ and warming had opposite effects on these genes. The signal intensities of Pcc and Rubisco genes were significantly higher (*P* < 0.05) under eCO_2_, but relatively lower under warming compared to ambient. However, the combination of eCO_2_ and warming also showed a significantly (*P* < 0.01) positive effect on these two genes. These results suggested that eCO_2_ and eCO_2_ + warming potentially increased carbon fixation.

The signal intensities of genes involved in methane production and oxidation showed different patterns in response to three treatments. Elevated CO_2_ alone significantly (*P* < 0.05) increased the signal intensities of *mcrA* for CH_4_ production and *pmoA* for CH_4_ oxidation, while the signal intensities of these two genes decreased under warming at marginally significant (*P* = 0.085) or significant (*P* = 0.033) levels, respectively (**Supplementary Figure S5**). The combination of eCO_2_ and warming significantly (*P* < 0.05) increased the signal intensities of *mcrA*, but had no effect on *pmoA*. These results indicate that warming may have negative effects on soil methane metabolism, while eCO_2_ had significant positive effects. When combined, warming may, to some extent, counteract the positive effects of eCO_2_.

**FIGURE 2:**
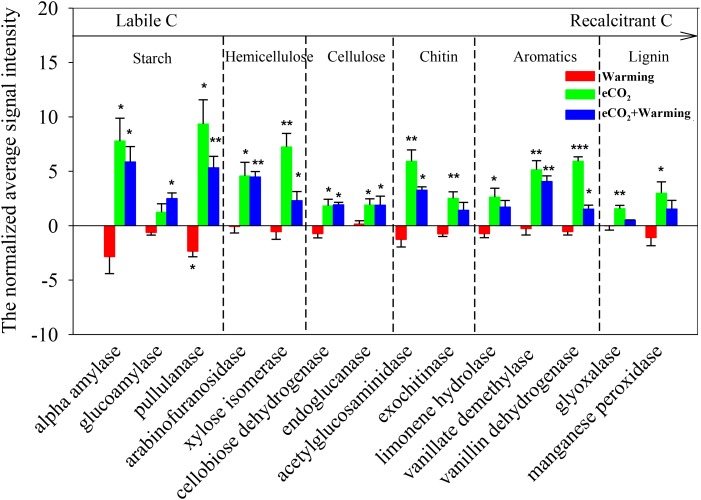
Significant differences of detected genes involved in C degradation in response to treatments. All data are presented as differences of mean (treatment-ambient) ± standard errors (SEs). Significant differences were calculated by *t*-tests and marked by asterisks. ^∗∗∗^*P* ≤ 0.001, ^∗∗^*P* ≤ 0.01, ^∗^*P* ≤ 0.05.

Notably, genes involved in C degradation were also dramatically affected by all three treatments (**Figure 2**). Among these, only the signal intensities of genes encoding pullulanase for starch degradation decreased by a significant (*P* < 0.05) level under warming alone. However, eCO_2_ alone significantly (*P* < 0.05) increased the signal intensities of functional genes for degradation of both labile C (starch, hemicellulose, cellulose and chitin) and recalcitrant C (aromatic and lignin) (Zhou et al., 2011; Xue et al., 2016a), including those encoding alpha amylase and pullulanase for starch decomposition, arabinofuranosidase and xylose isomerase for hemicellulose decomposition, cellobiose dehydrogenase and endoglucanase for cellulose decomposition, acetylglucosaminidase and exochitinase for chitin decomposition, limonene hydrolase, vanillate demethylase, and vanillin dehydrogenase for aromatic component degradation, glyoxalase and manganese peroxidase for lignin decomposition. The combination of eCO_2_ and warming significantly increased (*P* < 0.05) the signal intensities of most of the functional genes involved in the degradation of labile C. These results revealed that eCO_2_ had a dramatically positive effect on labile and recalcitrant C degradation, while warming likely had a relatively strong offset effect on the genes involved in degradation of recalcitrant C, especially for lignin-degradation genes, whereas the signal intensities of these genes had no significant change under warming alone.

**FIGURE 3:**
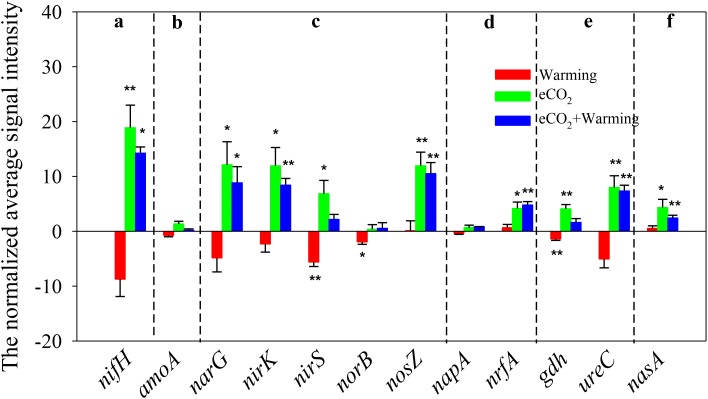
Significant differences of detected genes involved in the N cycle under Warming, eCO_2_, eCO_2_ + Warming treatments. **(a)** N_2_ fixation; **(b)** Nitrification; **(c)** Denitrification; **(d)** Dissimilatory N reduction to ammonium; **(e)** Ammonification; **(f)** Assimilatory N reduction. All data are presented as differences of mean (treatment-ambient) ± standard errors (SEs). Significant differences were calculated by *t*-tests and marked by asterisks. ^∗∗∗^*P* ≤ 0.001, ^∗∗^*P* ≤ 0.01, ^∗^*P* ≤ 0.05.

There were 126 ± 7, 96 ± 12, 211 ± 22, and 188 ± 10 genes involved in N cycling detected under ambient, warming, eCO_2_ and eCO_2_ + warming treatments, respectively (**Supplementary Table S1**). Elevated CO_2_ significantly (*P* < 0.05) increased the signal intensity of genes involved in N_2_ fixation (*nifH*), denitrification (*narG, nirS/K* and *nosZ*), dissimilatory N reduction to ammonium (*nrfA*), ammonification (*gdh* and *ureC*) and assimilatory N reduction (*nasA*), while warming significantly (*P* < 0.05) decreased the signal intensity of *nirS, nosB* (denitrification) and *gdh* (**Figure 3**). In addition, among the 13 functional genes detected in N cycling, 7 were stimulated significantly under the eCO_2_ + warming treatment, including *nifH, narG, nirK, nosZ, nrfA, ureC* and *nasA*. The signal intensities of *nirS* and *gdh* were significantly enhanced (*P* < 0.05) under eCO_2_ and suppressed (*P* < 0.05) under warming, while they remained unchanged under eCO_2_ + warming. These results suggest that eCO_2_, either alone or in combination with warming, may have a positive effect on soil N cycling by increasing the abundance of functional genes, though for some genes the effect was counteracted by warming.

Two functional genes involved in P cycling were detected by GeoChip, exopolyphosphatase (Ppx) for inorganic polyphosphate degradation and polyphosphate kinase (Ppk) for polyphosphate biosynthesis in prokaryotes (**Supplementary Figure S6**). The signal intensity of Ppk was significantly increased (*P* < 0.05) under eCO_2_, and the signal intensity of Ppx was significantly decreased (*P* < 0.05) under warming. The combination of warming and eCO_2_ had no apparent effect on these two genes.

### Linkages Between Microbial Community Structure and Soil Properties

**FIGURE 4:**
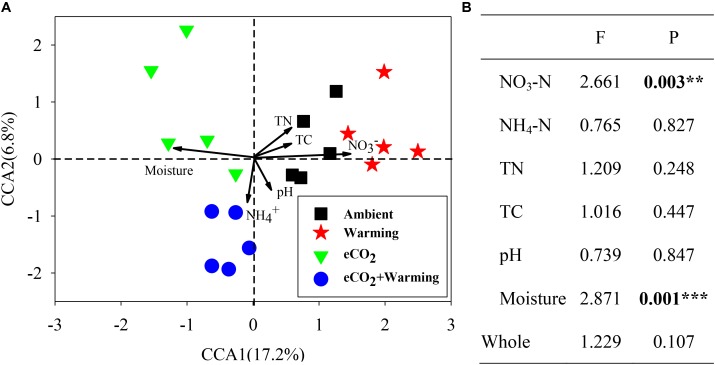
Canonical correspondence analysis (CCA) of GeoChip data and soil properties **(A)**. Model significances **(B)**. Asterisks denote the *P*-value for the difference: ^∗∗∗^*P* ≤ 0.001, ^∗∗^*P* ≤ 0.01.

To investigate the relationship between microbial community structure and soil properties (NO_3_-N, NH_4_-N, TN, TC, pH and moisture), a canonical correspondence analysis (CCA) was performed (**Figure 4A**). The communities from ambient, eCO_2_ + warming treatments separated clearly along the first canonical axis. Among these soil properties only NO_3_-N and moisture significantly (*P* < 0.01) correlated with all detected genes (**Figure 4B**), while other soil properties showed significant correlations with individual functional genes. The correlation between individual functional genes involved in C, N and P cycling and soil properties were further analyzed by the Mantel test. In total, 9, 2, 2, 25, and 3 genes involved in C and N cycling significantly (*P* < 0.05) correlated with soil NO_3_-N, TN, TC, moisture, and all soil properties, respectively (**Supplementary Table S2**). For example, genes involved in C degradation (*amyA*, isopullulanase, *pulA, ara, xylA, CDH*, acetylglucosaminidase, exochitinase, pectinase, *vanA, vdh, mnp*), C fixation (Pcc and Rubisco), methane metabolism (*mcrA and pmoA*), N fixation (*nifH*), ammonification (*gdh* and *ureC*), denitrification (*narG, nirK*/S, *nosB, nosZ*) and P cycling (Ppk and Ppx) were significantly (*P* < 0.05) correlated with soil moisture. In addition, the genes involved in C degradation (*ara, CDH*, acetylglucosaminidase), C fixation (Pcc), methane metabolism (*mcrA* and *pmoA*), N fixation (*nifH*), ammonification (*ureC*) and P cycling (Ppx) were significantly (*P* < 0.05) correlated with NO_3_-N. These results indicated that NO_3_-N and moisture may be the main environmental factors influencing the microbial functional structure in this grassland.

## Discussion

Soil microbial communities regulate many biogeochemical processes (e.g., C, N cycling) in response to global climate change, which in turn shape ecosystem functions (Castro et al., 2010). Here, we conducted a multi-factor experiment for climate change in a warmed semi-arid grassland to evaluate how these factors (warming, eCO_2_ and their combined effect) impact soil microbial communities. By using GeoChip, our results demonstrated that the composition and functional structure of the communities shifted substantially under warming, eCO_2_, and eCO_2_ + warming treatments. In addition, key functional genes involved in C, N, and P cycling produced distinct changes under the different treatments and were significantly correlated with soil properties. This study gives new insights into microbial responses and feedbacks to global climate change in grasslands.

### Treatment Effects on Microbial Communities Structures

At this experimental site the composition and phylogenetic and functional structures of soil microbial communities were dramatically altered under warming, eCO_2_, and eCO_2_ + warming. Previous studies have shown that microbial community structure shifted under eCO_2_ (He et al., 2010b, 2014; Yu et al., 2018) and warming (Sheik et al., 2011; Xue et al., 2016a,b). Our results agreed with these reports, which were supported by both the Adonis and DCA analysis of all detected genes. Moreover, the relative abundance of functional genes derived from five dominant phyla was also significantly altered, suggesting that the abundances of these microorganisms may increase under both eCO_2_ and eCO_2_ + warming. In this water-constrained grassland, eCO_2_ increased soil water availability by inducing the leaf transpiration of plant and increasing plant water-use efficiency (Morgan et al., 2004, 2011), while warming had the opposite effect (Reyes-Fox et al., 2014; Zelikova et al., 2014). In compared with ambient, soil moisture significantly (*P* = 0.039, *t*-test) decreased under warming alone, but greatly increased under both eCO_2_ (*P* = 0.024, *t*-test) and eCO_2_ + warming (*P* = 0.066, *t*-test) treatments, suggesting that the eCO_2_-induced water conserving effects may be greater than the desiccating effects of the warming-induced in this semiarid grassland (**Table 1**). These results agree with the previous report from this site (Carrillo et al., 2014). Previous studies of the BioCON site demonstrated that eCO_2_ significantly increased soil pH and moisture as well as shifted the functional and phylogenetic composition and structure of microbial communities in a grassland ecosystem (He et al., 2010b; Deng et al., 2012). In addition, a multifactor warming experiment showed that warming and added precipitation altered the soil microbial community composition in a grass prairie (Castro et al., 2010). Most importantly, the combined effects of eCO_2_ and warming were also significant for both total functional genes and *gyrB* genes by Adonis analysis, implying significant impacts by eCO_2_ and warming on the soil microbial community.

### Warming Effect on Functional Genes

How soil microbial functional processes (e.g., C, N, and P dynamics) will respond to climate change is critical issue for PHACE studies. In our results, the abundance and diversity of functional genes involved in functional processes were modified under warming. Several previous studies showed inconclusive responses by soil microbial communities under warming. For example, some key metabolic pathways, such as labile C degradation and nitrogen cycling, were enriched under warming (Zhou et al., 2011; Luo et al., 2014), or altered (increased or decreased) depending on the individual gene (Xue et al., 2016a). Moreover, some experimental sites found declines in microbial biomass respiration and carbon degradation processes within microbial communities in response to warming (Allison and Treseder, 2008; Allison et al., 2010; Romero-Olivares et al., 2017). A previous study also showed that the abundance of genes associated with C and N cycling decreased with warming in a Tibetan grassland (Yue et al., 2015). Those findings are generally consistent with the results presented here, in which the signal intensities of 13 genes involved in carbon degradation decreased under warming, though the differences were significant for only one gene (pullulanase), suggesting a relatively weak effect of warming on soil C dynamics. In addition, the signal intensities of 18 genes involved in C fixation, methane metabolism, N cycling, and P cycling were also decreased under warming, especially for 5 genes (*pmoA, nirS, norB, gdh*, and Ppx) which showed a significant (*P* < 0.05) decrease. These phenomena could be attributed to the fact that warming decreases soil water availability in this semiarid grassland (**Table 1**), which may suppress soil microbial activity and microbial functional processes (Allison and Treseder, 2008). Moreover, the microorganisms may harbor one gene which could also harbor the other genes catalyzing the processes involved in denitrification. Experimental warming often increases soil microbial functional processes in water unconstrained ecosystems (Zhou et al., 2011), however, soil water availability is a limiting factor for biological activity in this semiarid grassland (Dijkstra et al., 2010). This inference is also supported by our Mantel test, showing that many of the functional genes involved C, N, and P cycling have significant (*P* < 0.05) correlation with soil moisture (**Supplementary Table S2**). In addition, the significant decrease in abundance of *nirS* and *norB* may lead to an inhibition of microbial denitrification processes, and accordingly we also found a relatively higher concentration of soil nitrate under warming than under ambient (**Table 1** and **Figure 3**). Moreover, the abundance of *pmoA* genes significantly decreased, suggesting that CH_4_ uptake may reduce under warming. Although the CH_4_ flux was not measured in this study, the inference was confirmed by a previous study of this PHACE site (Dijkstra et al., 2013b). Results of the current study revealed a possible weak negative microbial feedback to warming in this semiarid grassland.

### Elevated CO_2_ Effect on Functional Genes

Elevated CO_2_ stimulated microbial functional processes and relevant soil functions. A study of this PHACE experimental site showed a positive feedback of microbial communities under eCO_2_ (Nie et al., 2013), while other reports showed that eCO_2_ has no significant response (Sinsabaugh et al., 2003; Austin et al., 2009) at the FACE site. Additionally, several previous studies showed that key genes involved in C degradation, C fixation, and methane metabolism cycling were stimulated under eCO_2_ in grassland, agricultural, and forest ecosystems (He et al., 2010b; Xiong et al., 2015; Yu et al., 2018). These results appear consistent with the present study, using the same GeoChip technology, revealing that the abundances of most of the functional gene involved in C cycling were significantly enhanced under eCO_2_. The effect of eCO_2_ on soil microbial communities possibly occurs via altered soil properties (e.g., pH and moisture) and increased C allocation to fine roots (He et al., 2010b; Morgan et al., 2011). However, in this water constrained ecosystem, the decomposition and plant inputs to soil may be regulated by soil water availability (Carrillo et al., 2011). In the current study, the signal intensities of 13 genes involved in both labile and recalcitrant C degradation were significantly increased, suggesting that microbial C decomposition may be stimulated under eCO_2_. The C fixation process was also enhanced by the significant increase of Pcc and Rubisco gene abundances, which is probably involved in the microbial community mediation response strategy to the gradual decrease in soil organic C due to faster decomposition (Carrillo et al., 2011). The decrease of soil total carbon has been observed not only in this PHACE site, but also in an agricultural FACE site (Xiong et al., 2015). The total signal intensities of *mcrA* and *pmoA* genes were significantly enhanced under eCO_2_, which is in agreement with previous studies of forest and agricultural FACE sites (Xiong et al., 2015; Yu et al., 2018). The methane production may be stimulated under eCO_2_, which could enhance methane uptake by increasing substrate availability for the methanotrophs. Moreover, this was also supported by a study at this PHACE site, showing that CH_4_ uptake was enhanced by increased soil moisture under eCO_2_ (Dijkstra et al., 2013b). eCO_2_ not only impacted soil C cycling driven by belowground microorganisms, but also altered the soil microbial N cycling process. The current study showed that the signal intensities of most N cycling genes (e.g., *nifH, nrfA, gdh, ureC, nasA, narG, nirK/S, nosZ*) were significantly increased under eCO_2_. This is most likely due to the fact that the greater soil water availability and C inputs from eCO_2_ may enhance the soil microbial activity and N demand (Carrillo et al., 2012; He et al., 2014). In addition, this conclusion was supported by soil properties data which showed a significant decrease of soil NO_3_-N, NH_4_-N under eCO_2_ (**Table 1**). Consequently, our results showed a potentially positive microbial response to eCO_2_.

### Elevated CO_2_ + Warming Effect on Functional Genes

The combined effects of eCO_2_ and warming altered microbial functional processes in a manner similar to eCO_2_ alone. It has been previously shown that warming can offset the positive effects of eCO_2_ on soil water availability in this PHACE site (Carrillo et al., 2014; Reyes-Fox et al., 2014). Consistent with these studies, the soil moisture under eCO_2_ + warming was lower than under eCO_2_ alone, but was marginally (*P* = 0.066) higher than ambient conditions (**Table 1**). However, whether the combination of eCO_2_ and warming had similar effects on soil microbial functional processes remains unknown. In the present study, the signal intensities of genes involved in labile C degradation were significantly increased under eCO_2_ + warming treatment, but for some recalcitrant C degradation genes (limonene hydrolase, glyoxalase, and manganese peroxidase) the changes were not significant (**Figure 2**). In comparison with the effect of eCO_2_ alone, the offset of warming was relatively weak for soil labile C, but comparatively strong for soil recalcitrant C dynamics. These phenomena could be explained by a previous study of this site, showing that the labile C pool size was greatly altered under eCO_2_ + warming in 2008 due to the increase in C input mediated by soil water availability (Carrillo et al., 2011). The signal intensities of two genes involved in C fixation were significantly enhanced under both eCO_2_ and eCO_2_ + warming treatments, indicating that eCO_2_ may have a robust effect on C fixation processes (**Supplementary Figure S4**). In addition, a significantly higher signal intensity of *mcrA* for methane production was observed with the eCO_2_ + warming treatment (**Supplementary Figure S5**). We speculate that methanogenic activity was promoted by the large input of labile carbon (Wachinger et al., 2000; Knorr et al., 2008). For N cycling, 7 and 9 genes abundances were significantly increased under eCO_2_ + warming or eCO_2_ alone treatments, respectively. These results potentially suggest that eCO_2_ + warming has a relatively positive effect on soil microbial functional process, although warming, to some extent, offset the priming effect of eCO_2_. Our results provide support to previous studies that suggested the response of soil processes to eCO_2_ + warming are more similar to those of eCO_2_ alone than of warming alone (Dieleman et al., 2012; Nie et al., 2013).

This study demonstrated that microbial community structure and functional processes were altered in response to climate change in this semiarid grassland ecosystem. Our results highlight three major mechanisms by which microbial communities could regulate soil microbial functional processes in response to global climate change. eCO_2_ had strong positive effects on microbial communities by increasing the microbial functional diversity and soil microbial C and N cycling, while warming had a weak negative effect on microbial communities. The combination of eCO_2_ and warming induced a relatively positive feedback from microbial communities although warming offset part of the priming effect caused by eCO_2_. However, this study only examined microbial communities in single season of a year that might not reflect the changes of all microorganisms. Our future study may focus on the temporal dynamics of soil microbial communities in response to multiple climate change factors with the substantiation of actual process measurements.

## Author Contributions

All authors listed have made a substantial, direct and intellectual contribution to the work, and approved it for publication.

## Conflict of Interest Statement

The authors declare that the research was conducted in the absence of any commercial or financial relationships that could be construed as a potential conflict of interest.

## Footnotes

1 http://ieg.ou.edu/microarray/

2 http://mem.rcees.ac.cn/download.html

3 www.r-project.org
