# Supplementary material for: Elevated CO2 and Warming Altered Grassland Microbial Communities in Soil Top-Layers
Source: Front Microbiol. 2018 Aug 14;9:1790. doi: 10.3389/fmicb.2018.01790 (PMC6102351; doi:10.3389/fmicb.2018.01790)
Supplement: Supplementary file 1 [file Data_Sheet_1.PDF]

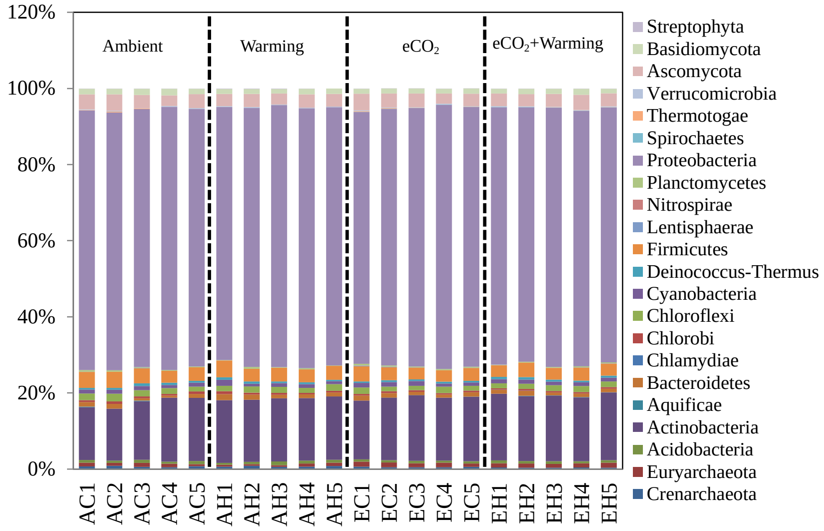

Figure S1. Taxonomic composition of the soil microbial community under different treatments detected by GeoChip at phylum level.
