# Supplementary material for: Elevated CO2 and Warming Altered Grassland Microbial Communities in Soil Top-Layers
Source: Front Microbiol. 2018 Aug 14;9:1790. doi: 10.3389/fmicb.2018.01790 (PMC6102351; doi:10.3389/fmicb.2018.01790)
Supplement: Supplementary file 2 [file Data_Sheet_2.PDF]

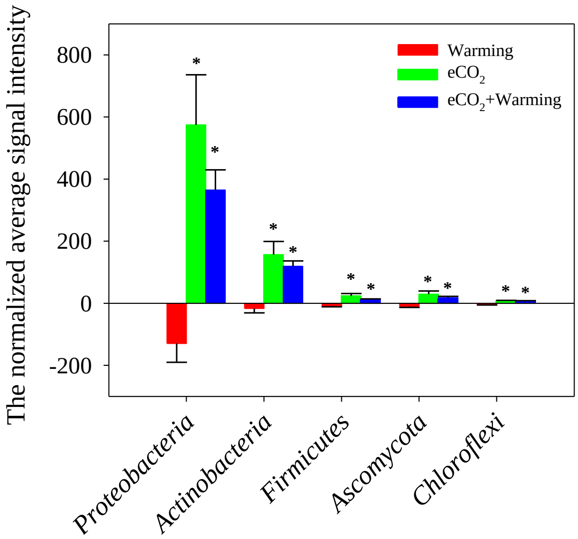

Figure S2. Relative abundance of genes derived from dominant phylum. All data are presented as differences of mean (treatment-ambient) ± standard errors (SEs). *:$P<0.05$, based on Student’s t test.
