# Supplementary material for: Elevated CO2 and Warming Altered Grassland Microbial Communities in Soil Top-Layers
Source: Front Microbiol. 2018 Aug 14;9:1790. doi: 10.3389/fmicb.2018.01790 (PMC6102351; doi:10.3389/fmicb.2018.01790)
Supplement: Supplementary file 3 [file Data_Sheet_3.PDF]

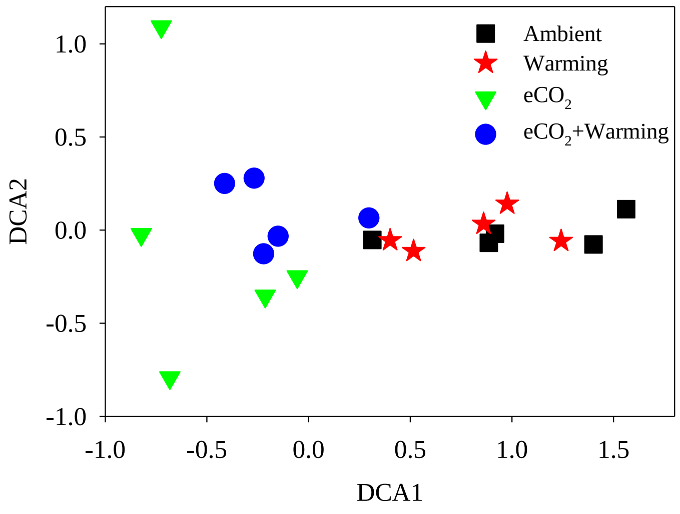

Figure S3. Detrended correspondence analysis (DCA) of all detected *gyrB* genes across all treatments
