# Supplementary material for: Elevated CO2 and Warming Altered Grassland Microbial Communities in Soil Top-Layers
Source: Front Microbiol. 2018 Aug 14;9:1790. doi: 10.3389/fmicb.2018.01790 (PMC6102351; doi:10.3389/fmicb.2018.01790)
Supplement: Supplementary file 4 [file Data_Sheet_4.PDF]

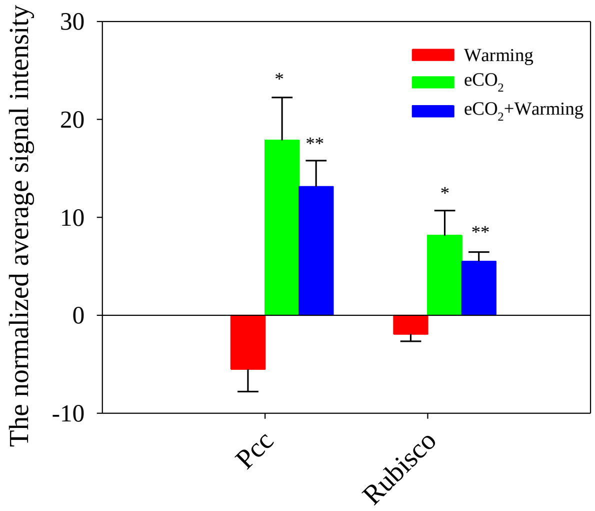

Figure S4. Significant differences of detected genes involved in carbon fixation in response to treatments. Pcc: propionyl-CoA carboxylase; Rubisco: ribulose-1,5-bisphosphate carboxylase/oxygenase. Error bars represent standard error of the mean (treatment-ambient). Significance among the treatments was calculated by *t* tests and marked by asterisks. **:$P \leq 0.01$, *:$P \leq 0.05$.
