# Supplementary material for: Elevated CO2 and Warming Altered Grassland Microbial Communities in Soil Top-Layers
Source: Front Microbiol. 2018 Aug 14;9:1790. doi: 10.3389/fmicb.2018.01790 (PMC6102351; doi:10.3389/fmicb.2018.01790)
Supplement: Supplementary file 7 [file Table_1.DOCX]

**Table S1.** Number of detected key functional genes involved in carbon, nitrogen, phosphorus, and sulfur cycling.

| Gene category | No. ± SEM of genes | | | |
| --- | --- | --- | --- | --- |
|  | Ambient | Warming | eCO_2_ | eCO_2_+Warming |
| C cycling | 138±9^bc^ | 94±12^c^ | 245±26^a^ | 200±9^ab^ |
| Acetogenesis | 1±0 | 1±0 | 2±1 | 1±0 |
| C degradation | 96±6^bc^ | 66±9^c^ | 163±17^a^ | 135±7^ab^ |
| C fixation | 33±3^bc^ | 24±3^c^ | 63±8^a^ | 54±4^ab^ |
| Methan | 8±1^bc^ | 4±1^c^ | 16±1^a^ | 10±1^b^ |
| N cycling | 126±7^b^ | 96±12^b^ | 211±22^a^ | 188±10^a^ |
| Ammonification | 15±2^b^ | 9±2^b^ | 28±3^a^ | 24±1^a^ |
| Assimilatory N reduction | 1±0^b^ | 1±1^b^ | 5±2^a^ | 3±1^ab^ |
| Denitrification | 65±3^bc^ | 51±6^c^ | 109±13^a^ | 96±8^ab^ |
| Dissimilatory N reduction | 6±1^b^ | 7±1^b^ | 11±1^a^ | 12±1^a^ |
| Nitrification | 1±0 | 1±0 | 3±1 | 1±0 |
| N fixation | 38±4^bc^ | 28±3^c^ | 55±4^a^ | 51±1^ab^ |
| P utilization | 39±2 | 32±2 | 43±5 | 38±1 |
| S cycling | 56±4^ab^ | 41±6^b^ | 72±8^a^ | 64±3^ab^ |
| Total | 1269±78^bc^ | 1058±99^c^ | 2217±269^a^ | 1882±106^ab^ |

Significance among the treatments was calculated by multi-way analysis of variance (ANOVA) at *P*<0.05 level and marked with a, b and c.
