# Supplementary material for: Elevated CO2 and Warming Altered Grassland Microbial Communities in Soil Top-Layers
Source: Front Microbiol. 2018 Aug 14;9:1790. doi: 10.3389/fmicb.2018.01790 (PMC6102351; doi:10.3389/fmicb.2018.01790)
Supplement: Supplementary file 8 [file Table_2.DOCX]

**Table S2.** Significantly correlated between signal intensities of functional genes involved in C and N cycling and soil properties analyzed by Mantel analysis.

| Gene/enzyme | Functional process | NO_3_-N | NH_4_-N | TN | TC | Moisture | pH | All factors |
| --- | --- | --- | --- | --- | --- | --- | --- | --- |
| *amyA* | C degradation | 0.116 | 0.262 | 0.653 | 0.775 | **0.012*** | 0.245 | 0.26 |
| isopullulanase | C degradation | 0.491 | 0.412 | 0.988 | 0.959 | **0.044*** | 0.294 | 0.596 |
| *pulA* | C degradation | 0.133 | 0.603 | 0.768 | 0.778 | **0.035*** | 0.397 | 0.565 |
| *ara* | C degradation | **0.001***** | 0.37 | 0.221 | 0.264 | **0.001***** | 0.677 | **0.014*** |
| *xylA* | C degradation | 0.052• | 0.862 | 0.706 | 0.765 | **0.004**** | 0.448 | 0.417 |
| *CDH* | C degradation | **0.021*** | 0.589 | **0.011*** | 0.073• | **0.039*** | 0.589 | 0.054• |
| acetylglucosaminidase | C degradation | **0.021*** | 0.721 | 0.578 | 0.536 | **0.001***** | 0.712 | 0.196 |
| exochitinase | C degradation | 0.217 | 0.774 | 0.687 | 0.716 | **0.001***** | 0.716 | 0.531 |
| pectinase | C degradation | 0.135 | 0.415 | 0.528 | 0.804 | **0.008**** | 0.556 | 0.333 |
| *vanA* | C degradation | 0.362 | 0.739 | 0.682 | 0.896 | **0.019*** | 0.294 | 0.546 |
| *vdh* | C degradation | 0.068• | 0.322 | 0.694 | 0.683 | **0.002**** | 0.5 | 0.309 |
| *mnp* | C degradation | 0.373 | 0.764 | 0.676 | 0.648 | **0.017*** | 0.78 | 0.698 |
| Pcc | C fixation | **0.049*** | 0.808 | 0.849 | 0.95 | **0.006**** | 0.406 | 0.546 |
| Rubisco | C fixation | 0.603 | 0.615 | 0.96 | 0.959 | **0.025*** | 0.363 | 0.75 |
| *mcrA* | Methane production | **0.012*** | 0.521 | 0.356 | 0.377 | **0.001***** | 0.566 | 0.061• |
| *pmoA* | Methane oxidation | **0.008**** | 0.601 | 0.202 | 0.212 | **0.009**** | 0.721 | 0.176 |
| *nifH* | Nitrogen fixation | **0.002**** | 0.85 | 0.149 | 0.175 | **0.002**** | 0.428 | **0.034*** |
| *gdh* | Ammonification | 0.397 | 0.13 | 0.963 | 0.951 | **0.027*** | 0.348 | 0.422 |
| *ureC* | Ammonification | **0.023**** | 0.712 | 0.455 | 0.599 | **0.002**** | 0.328 | 0.242 |
| *narG* | Denitrification | 0.589 | 0.529 | 0.955 | 0.963 | **0.036*** | 0.344 | 0.768 |
| *nirK* | Denitrification | 0.096• | 0.395 | 0.291 | 0.336 | **0.026*** | 0.256 | 0.2 |
| *nirS* | Denitrification | 0.257 | 0.569 | 0.29 | 0.358 | **0.045*** | 0.245 | 0.274 |
| *norB* | Denitrification | 0.107 | 0.463 | 0.116 | **0.039*** | 0.107 | 0.548 | 0.103 |
| *nosZ* | Denitrification | 0.076• | 0.212 | 0.604 | 0.75 | **0.014*** | 0.146 | 0.126 |
| Ppk | P utilization | 0.112 | 0.565 | 0.577 | 0.795 | **0.002**** | 0.188 | 0.205 |
| Ppx | P utilization | **0.001***** | 0.713 | **0.003**** | **0.004**** | **0.001***** | 0.859 | **0.002**** |

Bold P values indicate statistical significance (*P* <0.05). Asterisks denote the *P*-value for the difference. ***:*P*≤0.001, **:*P*≤0.01, *:*P*≤0.05, •: *P*<0.1 based on Mantel analysis.
